# Can HRCT be used as a marker of airway remodelling in children with difficult asthma?

**DOI:** 10.1186/1465-9921-7-46

**Published:** 2006-03-27

**Authors:** S Saglani, G Papaioannou, L Khoo, M Ujita, PK Jeffery, C Owens, DM Hansell, DN Payne, A Bush

**Affiliations:** 1 Respiratory Paediatrics, Royal Brompton Hospital, London, UK; 2 Department of Radiology, Great Ormond Street Hospital, London, UK; 3 Department of Radiology, Royal Brompton Hospital, London, UK; 4 Lung Pathology, Imperial College London at the Royal Brompton Hospital, London, UK

## Abstract

**Background:**

Whole airway wall thickening on high resolution computed tomography (HRCT) is reported to parallel thickening of the bronchial epithelial reticular basement membrane (RBM) in adult asthmatics. A similar relationship in children with difficult asthma (DA), in whom RBM thickening is a known feature, may allow the use of HRCT as a non-invasive marker of airway remodelling. We evaluated this relationship in children with DA.

**Methods:**

27 children (median age 10.5 [range 4.1–16.7] years) with DA, underwent endobronchial biopsy from the right lower lobe and HRCT less than 4 months apart. HRCTs were assessed for bronchial wall thickening (BWT) of the right lower lobe using semi-quantitative and quantitative scoring techniques. The semi-quantitative score (grade 0–4) was an overall assessment of BWT of all clearly identifiable airways in HRCT scans. The quantitative score (BWT %; defined as [airway outer diameter – airway lumen diameter]/airway outer diameter ×100) was the average score of all airways visible and calculated using electronic endpoint callipers. RBM thickness in endobronchial biopsies was measured using image analysis. 23/27 subjects performed spirometry and the relationships between RBM thickness and BWT with airflow obstruction evaluated.

**Results:**

Median RBM thickness in endobronchial biopsies was 6.7(range 4.6 – 10.0) μm. Median qualitative score for BWT of the right lower lobe was 1(range 0 – 1.5) and quantitative score was 54.3 (range 48.2 – 65.6)%. There was no relationship between RBM thickness and BWT in the right lower lobe using either scoring technique. No relationship was found between FEV_1 _and BWT or RBM thickness.

**Conclusion:**

Although a relationship between RBM thickness and BWT on HRCT has been found in adults with asthma, this relationship does not appear to hold true in children with DA.

## Background

Thickening of the epithelial reticular basement membrane (RBM) is one characteristic feature of airway remodelling in asthma. It has been reported in both adults and school-aged children [1-3]. However, the clinical significance of RBM thickening, and the mechanisms involved in its pathogenesis remain unclear. In particular, it is not known at what age RBM thickening begins.

RBM thickness can be measured in endobronchial biopsy (EB), but this requires an invasive procedure, and the opportunities for obtaining EB in children are therefore limited. The potential to investigate the timing and natural history of RBM thickening, and other features of airway remodelling in children, would be increased by the development of non-invasive techniques, thus providing the opportunity to monitor changes over time and in response to treatment. A number of non-invasive techniques have been developed for the study of airway inflammation in asthma [4]. In comparison, there has been little interest in the development of similar techniques to study airway structural changes. One exception is the use of high-resolution computed tomography (HRCT) to study airway wall changes in asthma [5]. Bronchial wall thickening (BWT) on HRCT has been shown to be a consistent finding in children with difficult asthma [6] and a relationship between BWT and RBM thickness has been demonstrated in adults with asthma, following treatment with oral corticosteroids and short-acting β_2_-agonists [7]. The demonstration of a similar relationship in children with difficult asthma would therefore allow HRCT to be used as a surrogate marker of RBM thickening.

Airway remodelling is often considered to contribute to the element of irreversible airflow obstruction, which is a feature of some patients with asthma. Kasahara and colleagues reported a significant negative correlation between post-bronchodilator forced expiratory volume in one second (FEV_1_) and both BWT on HRCT and RBM thickness in EB [7]. However, other cross-sectional studies have failed to demonstrate an association between FEV_1 _and either BWT [6,8] or RBM thickness [3,9].

The aims of the present study were therefore to investigate i) whether BWT, as shown on HRCT, can be used as a non-invasive indicator of RBM thickness in EB, in a group of children with difficult asthma, and ii) the association between the degree of airflow limitation, assessed by FEV_1 _and BWT or RBM thickness.

## Methods

### Subjects

Twenty-seven children (median age 10.5 [range 4.1–16.7] years) with difficult asthma, who underwent bronchoscopy, EB and HRCT between January 2000 and November 2002 were identified and studied retrospectively. Subjects underwent bronchoscopy, bronchoalveolar lavage and EB as part of their clinical assessment in order to help confirm the diagnosis of asthma and to exclude any other associated abnormalities such as structural airway abnormalities or significant infection. They underwent HRCT to exclude bronchiectasis or any other airways disease such as obliterative bronchiolitis that may have been an alternative explanation for their disease severity. Difficult asthma was defined as persistent symptoms requiring rescue bronchodilator therapy > 3 days per week, despite ≥ 800 micrograms per day of inhaled budesonide (or equivalent), and long acting β_2 _agonists, and/or regular oral steroids. All subjects that had a bronchoscopy and EB in the defined time period were identified. Not all had a biopsy of sufficient quality (defined as a biopsy containing recognisable epithelium, RBM and subepithelium, with at least 1 mm of RBM) to quantify the RBM [3,10]. Therefore only those with a good quality biopsy were included in this study. There was no difference in age, sex or disease severity between subjects with and without good quality biopsies. The clinical details of subjects included are summarised in table 1.

**Table 1:** Clinical characteristics of children with difficult asthma

Number	27
Age*	10.5 (4.1 – 16.7)
Male/Female	17/10
FEV_1 _(% predicted)*, pre-bronchodilator ^a^	82.6 (32.1 – 118)
Atopic	21 (78%)
Treatment:	
Daily dose budesonide/equivalent*	2000 (800 – 4000) μm
Number on LABA	20 (74%)
Number on regular orals steroids	50(18.5%)

* median (range)

a – only 23/27 able to perform satisfactory spirometry

Twenty-three of 27 subjects also performed spirometry in accordance with American Thoracic Society guidelines [11]. Four subjects, aged between 4.1 and 5.2 years were unable to perform spirometry with a satisfactory technique. Sixteen of the 23 subjects that performed spirometry had received a 2-week course of oral corticosteroids before spirometry and 9 of those 16 performed spirometry before and after inhaled bronchodilator (short acting β_2 _agonist).

Informed parental consent was obtained prior to performance of EB and HRCT in all cases. Ethical approval was obtained to study all biopsies and HRCTs.

### Endobronchial biopsies

Flexible bronchoscopy was performed under general anaesthetic, as previously described [12]. Up to six EB were taken from the sub-carinae of the right lower lobe. Biopsies were fixed and processed into paraffin blocks. Step sections (5 μm thick) were cut 50 μm apart and stained with haematoxylin and eosin. At least one measurable section, which was well orientated and had identifiable epithelium, RBM and subepithelium, was chosen from each patient. If more than one biopsy, satisfying the above criteria, was obtained from the same patient, the between biopsy variability was assessed. RBM thickness was measured using computer-aided image analysis (NIH image 1.55; National Institute of Health, Bethesda, MD) as previously described [13]. Briefly, at a magnification of ×400, at least 40 measurements of RBM thickness were made 20 μm apart. A minimum length of 1 mm of RBM was assessed. The geometric mean of all measurements was calculated to represent thickness for that section. Measurements of RBM thickness were made without knowledge of the HRCT assessments.

### HRCT

All HRCTs were obtained at near total lung capacity with breath holding rehearsed before commencement of the HRCT. 1.5 mm thick sections were acquired at 10 mm intervals in the supine position using an electron beam ultrafast scanner (Imatron Inc., San Francisco, California) and images were reconstructed using a high spatial resolution reconstruction algorithm. Images were photographed using window settings optimised for paediatric lungs (centre: -500 H.U., width: 1500 H.U.) [14].

### HRCT scoring

A quantitative score that has previously been used to assess BWT in HRCT in adult asthmatics [7] was used. However, more recently a paediatric study comparing BWT in HRCT with endobronchial biopsies has used a semi-quantitative score [15]. Also, for clinical purposes, application of a quantitative score is time consuming. Therefore, in order to compare findings to previously published data and to assess whether there is any advantage in using a quantitative scoring system, both semi-quantitative and quantitative scores of BWT on HRCT were applied. HRCT images were assessed by two radiologists (LK, UM) for the semi-quantitative scoring and then by a third radiologist (GP) for the quantitative scoring; all radiologists were unaware of the clinical status of the subjects.

### Semi-quantitative score

HRCT images were assessed by two radiologists (LK, UM) independently. A semi-quantitative score for BWT in all sections of the HRCT was recorded. The evaluation of BWT was confined to clearly identifiable segmental and sub-segmental airways. A separate score was given to each lobe. Scores ranged from 0 to 4. 0 was normal wall thickness, 1 was minimal wall thickening, 2 was bronchial wall thickness half of the diameter of the adjacent blood vessel, 3 was bronchial wall thickness half to the same diameter of the adjacent vessel, and 4 was bronchial wall thickness greater than the diameter of the adjacent vessel. This score, which in the context of this study was only used to assess bronchial wall thickness has been used previously to assess the relationship between CT features of bronchiectasis and lung function [16]. Bronchial dilatation was not assessed. The mean of the two scores ascribed was used to assess the relationship between BWT and RBM thickness and FEV_1_.

### Quantitative score

**Figure 1:**
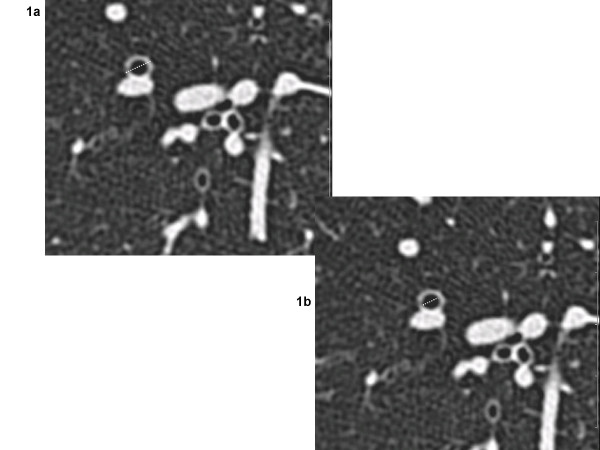
**Outer and inner bronchial diameters**. Magnified area of an axial HRCT of a child with difficult asthma showing a circular bronchus that was quantified. 1a) outer (Do = 0.5 cm) and 1b) inner (Di = 0.3 cm) bronchial diameters were measured as outlined.

**Figure 2:**
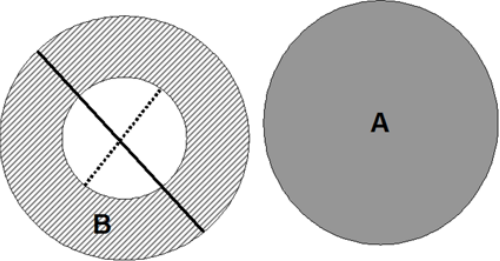
**Diagram of outer and inner diameters**. Diagramatic draft of the measurement techniques applied to magnified cross-sectional images. The obvious round-shaped artery (A) and bronchus (B) pairs were identified and the outer (Do) and inner (Di) diameter of the bronchus were measured (solid and dotted lines respectively). WT% was calculated as [(Do-Di)/Do] × 100.

The quantitative scoring system was based on that previously used in adult studies that have assessed BWT in HRCTs from asthmatics [7]. HRCT scans were loaded to a PACS workstation (mv1000, Siemens) and all images were analysed electronically. A magnification factor of 7 was applied in all images that were displayed in HRCT window settings. All clearly visible segmental and sub-segmental airway/vessel pairs that had a rounded cross-sectional circumference were measured manually by using electronic caliper endpoints (figures 1a and 1b). By estimating the Hounsfield units using the ROI (region of interest) tool, the endpoints were placed at the cut-off edge between the wall and the air. For each airway with an obvious circular appearance, the outer diameter was measured in the x and y axis. The shorter of these two, was termed the airway outer diameter (Do) (figure 1a). In the same axis, the airway inner diameter, or lumen diameter, (Di) was measured (figure 1b). The percentage of airway wall thickness (BWT) was calculated (BWT % = [Do-Di]/Do × 100) (figure 2).

### Statistical analysis

A weighted kappa coefficient was calculated to determine the level of agreement for the semi-quantitative score between the two HRCT observers [17]. The Kruskal-Wallis test was used to look for a relationship between numerical and categorical variables. The relationship between RBM thickness and % BWT, and RBM thickness and predicted FEV_1 _were assessed using Spearman's correlation (r_s_). The variability of RBM thickness within and between biopsies was calculated as the % coefficient of variation, by dividing the standard deviation of the measurements by the mean. All analyses were performed using the Statistical Package for the Social Sciences (SPSS) version 11.5.

## Results

Twenty-seven children had EB and HRCT performed no more than 4 months apart. Eighteen of 27 had both investigations on the same day (table 2). Median RBM thickness was 6.7 (range 4.6–10.0) μm. The coefficient of variation for within-biopsy measurements ranged from 1.6 – 7.4%, and that for variability between biopsies ranged from 6 – 21.6%.

**Table 2:** Patients who had investigations performed on same and different days

	HRCT & EB same day	HRCT & EB different days	Total
FEV_1 _available*	17	6	23
No FEV_1_	1	3	4
Total	18	9	27

* 10/17 had all 3 tests on the same day, 7/17 had spirometry on a different day

HRCT: high-resolution computed tomography

EB: endobronchial biopsy

FEV_1_: forced expiratory volume in 1 second

### Semi-quantitative BWT score and RBM thickness

**Figure 3:**
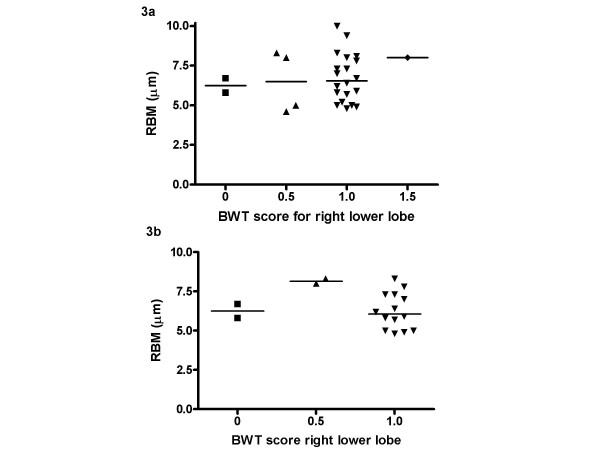
**RBM thickness and HRCT bronchial wall thickening using semi-quantitative score**. Relationship between RBM thickness in endobronchial biopsy and bronchial wall thickening on HRCT measured using a semi-quantitative score. 3a) all HRCTs and bronchial biopsies, 3b) only HRCTs and bronchial biopsies performed on the same day.

**Figure 5:**
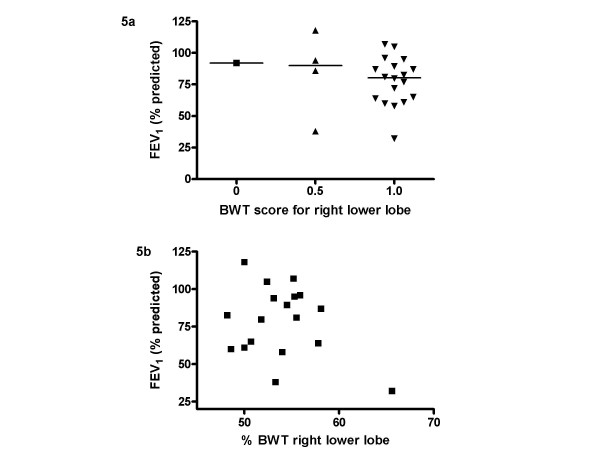
**HRCT bronchial wall thickening and FEV_1_**. 5a) Relationship between bronchial wall thickening on HRCT and FEV_1 _using a semi-quantitative score and 5b) using a quantitative scoring technique.

There was a moderate level of agreement between observers for HRCT scores for BWT of the right lower lobe (weighted κ = 0.54). The average of the two scores ascribed for the right lower lobe bronchus, near the site of EB, was used for subsequent analyses. Median score for BWT in the right lower lobe was 1 (range 0–1.5). None of the HRCTs had evidence of bronchiectasis. Subjects grouped according to BWT score showed there was no relationship between RBM thickness and median BWT score on HRCT scan (figure 3a). The result was the same when only those patients (18/27) who had EB and HRCT on the same day were included in the analysis (figure 3b). For the patients who also performed spirometry, median pre-bronchodilator FEV_1 _% predicted was 79.7% (range 32.1–118.0%). There was no difference in pre-bronchodilator FEV_1 _between the groups, based on BWT score (figure 5a).

### Quantitative BWT score and RBM thickness

**Figure 4:**
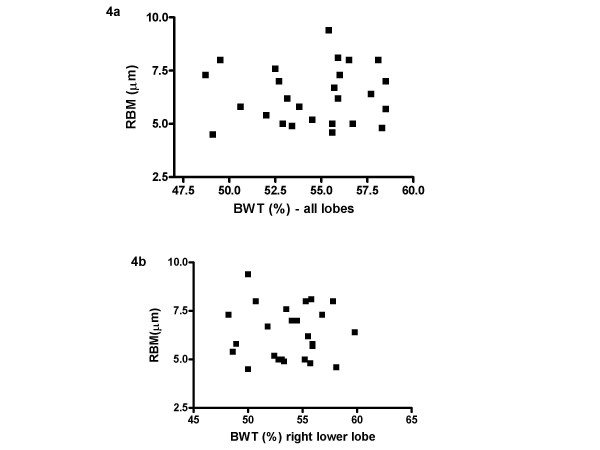
**RBM thickness and HRCT bronchial wall thickening using quantitative score**. Relationship between RBM thickness in endobronchial biopsy and 4a) bronchial wall thickening on whole HRCT and 4b) right lower lobe bronchial wall thickening, measured using a quantitative score.

A score was obtained for the average BWT for all lobes and also just for the right lower lobe (as this is where biopsies were taken). Median BWT for the whole scan was 55.5 (range 48.7 – 58.5)% and that for just the right lower lobe was 54.3 (range 48.2 – 65.6)%. There was no correlation between BWT for the whole scan and RBM thickness on EB (r_s _= 0.066, p = 0.75) (figure 4a) and there was also no relationship between BWT for the right lower lobe and RBM thickness (r_s _= 0.03, p = 0.89), (figure 4b). There was a good correlation between BWT score for the whole HRCT scan and that just for the right lower lobe (r_s _= 0.64, p < 0.001).

### RBM thickness, BWT and lung function

There was no relationship between pre-bronchodilator FEV_1 _and RBM thickness (r_s _= -0.155, p = 0.48). There was also no relationship between FEV_1 _and BWT on HRCT, measured using both techniques (figure 5a and 5b). 10/27 patients had HRCT, EB and spirometry on the same day (table 2). When they were analysed separately, there was no relationship between BWT and RBM thickness or pre-bronchodilator FEV_1_. Similarly, no relationship was seen between BWT and RBM or post-bronchodilator FEV_1 _when the 9 patients who had performed spirometry pre and post bronchodilator were analysed.

## Discussion

There was no relationship between RBM thickness in EB and BWT on HRCT in children with difficult asthma. Also, no relationship was found between FEV_1 _(% predicted) and BWT on HRCT or RBM thickness in EB.

In keeping with our previous findings from a group of children with difficult asthma, we found no relationship between RBM thickness and % predicted FEV_1 _[3]. Also, in agreement with Marchac and colleagues we found no relationship between BWT on HRCT and % predicted FEV_1_, nor did we find any evidence of bronchiectasis [6]. Our findings are in contrast to those of Kasahara and colleagues in adults [7] and de Blic and colleagues [15] in children.

A limitation of this study compared to that by Kasahara and colleagues in adults [7], was that there were no HRCT measurements from control subjects. However, given the ethical implications of unnecessary radiation exposure, it was not possible to justify performing HRCT in healthy children. It is especially important to consider measurements of both RBM thickness and BWT on HRCT in healthy children because of the influence of normal airway development in this age group [18]. However, a paediatric study that has reported a relationship between RBM thickness on EB and BWT on HRCT in difficult asthmatics also did not include healthy controls [15]. A further limitation of the current study was that patients were identified retrospectively, and lung function data was not available in all cases. This resulted in only a small number of patients with all data present. In some patients, tests (EB, HRCT and lung function) were not performed on the same day because the investigations were all clinically indicated and therefore were performed only when necessary.

As only 9/23 patients performed spirometry pre and post-bronchodilator, the relationship between BWT and lung function was assessed for all 23 patients using pre-bronchodilator FEV_1_. This is in contrast to the study by Kasahara and colleagues who compared post-bronchodilator FEV_1 _with BWT and RBM thickness [7], and may account for the discrepancy between the results of their study and the present one. When these 9 patients were analysed separately, no relationship was seen. However, the small number limits the ability to draw firm conclusions. In the present study, only 10/27 patients had HRCT, EB and spirometry performed on the same day (table 2). No relationship was found between any of the parameters when these patients were analysed separately, providing support for the results found for the group as a whole.

Two-thirds (18/27) of the subjects had HRCT and EB on the same day (table 2), while the remainder had a period of up-to 4 months between the tests. As EB was performed after a two-week course of oral steroids, it may be that this affected the results for those who had the tests separately. However, previous studies that have assessed the effect of steroid therapy on RBM thickness have shown a reduction in thickness after prolonged therapy for several months, not weeks [19], so a short course, even when given systemically is unlikely to have affected RBM thickness. Importantly, all subjects studied by Kasahara and colleagues did have pre-treatment with 2 weeks of prednisolone prior to HRCT in order to minimise the effects of any airway oedema. However, in the current study, when the patients who had both tests on the same day, immediately after completion of the steroid course, were analysed separately, there was still no relationship between BWT and RBM thickness.

In order to ensure that the failure to show a relationship between BWT and the other parameters was not due to the scoring technique used, and to ensure the scoring techniques used in previously published studies were used, [7,15] HRCTs were scored using both semi-quantitative [16] and quantitative techniques [7]. However, despite using 2 separate techniques and using independent observers to score the scans and ensuring an adequate level of agreement between observers for the semi-quantitative technique (weighted kappa > 0.5), there was still no relationship found between BWT and RBM thickness or FEV_1_. Furthermore, as the biopsies were taken from the right lower lobe, the CT score for that lobe alone was used in the analysis. It may be proposed that quantitative methods are more accurate than semi-quantitative scoring. However, we have demonstrated that with a moderate level of agreement between observers, the use of the semi-quantitative technique gives similar results to the quantitative technique. Although the quantitative technique might appear to be the more objective of the two, it also involves some degree of subjective bias, since the identification of the boundaries of the inner lumen and outer wall requires a judgement by the investigator. Importantly, there was a very good relationship between the quantitative BWT score for all lobes and that for just the right lower lobe, suggesting it may not be necessary to score all lobes for future studies.

Of note, in the present study, the median HRCT scores for BWT overall was only 1 (minimal wall thickening), suggesting that the extent of wall thickening was relatively mild. James and colleagues showed a relationship between RBM thickening and airway wall thickness in lung tissue obtained post-mortem from adults [20]. Therefore, it may be that unlike RBM thickening, whole airway wall thickening, as a reflection of remodelling, increases with age and is thus a later phenomenon. Data from Bai and colleagues, who found an increase in airway wall thickness in older, but not younger, subjects with fatal asthma would support this suggestion [21]. Furthermore, RBM thickening is only one structural airway change seen as part of the process of remodelling in asthma. Other changes such as adventitial thickening [21] or increase in smooth muscle [22], which have not yet been quantified in children with asthma [23], may contribute more to the thickness of the whole airway wall, and may occur later.

It might be proposed that a relationship was not found between RBM thickness and BWT because all patients included were relatively similar clinically, in terms of disease severity. They were all on high dose inhaled steroids and long acting beta agonists and despite this were still symptomatic on at least 3 days per week. Data concerning the relationship between RBM thickness and disease severity are controversial, whereby some have reported equal thickening in both mild and severe disease [3,24] whereas others have suggested a relationship between RBM thickness and disease severity [25]. This suggests that disease severity alone is unlikely to be the explanation for the lack of relationship between HRCT BWT and RBM thickness in the present study. However, a positive relationship between RBM thickness in EB and BWT on HRCT has been reported by de Blic and colleagues in a group of children with difficult asthma, all with similar disease severity [15]. Importantly, this was a weak relationship that could only be applied to the group as a whole. If individuals were considered, then even from their data BWT on HRCT cannot be used as a surrogate for RBM thickness on EB.

## Conclusion

In summary, these data demonstrate that measurements of BWT on HRCT cannot be used as a surrogate marker for RBM thickness in EB in children with difficult asthma. In addition, BWT measurements are not associated with the degree of airflow limitation in this group of patients.

## Competing interests

The author(s) declare that they have no competing interests.

## Authors' contributions

SS identified the subjects, analysed the biopsies, performed the data analysis, and prepared the manuscript. GP performed the quantitative HRCT measurements. LK and MU performed the semi-quantitative HRCT measurements. PKJ was involved in biopsy preparation and guided biopsy measurements. CO guided the quantitative HRCT measurements. DMH guided the semi-quantitative measurements. DNP and AB provided biopsies, and guided data analysis and manuscript preparation.
